# Dr. Harry Mayer Schall

**Published:** 1918-12

**Authors:** 


**Dr. Harry Mayer Schall,** Jefferson 1887, of Rochester, died in the General Hospital of that city, Oct. 9, of heart disease, aged 55.

